# A synthesis of modern organic carbon accumulation rates in coastal and aquatic inland ecosystems

**DOI:** 10.1038/s41598-018-34126-y

**Published:** 2018-10-24

**Authors:** Grace M. Wilkinson, Alice Besterman, Cal Buelo, Jessica Gephart, Michael L. Pace

**Affiliations:** 1 Department of Ecology, Evolution, and Organismal Biology, Iowa State University, IA 50011 Iowa, USA; 2 Department of Environmental Sciences, University of Virginia, Charlottesville, VA 22903 USA; 3 The National Socio-Environmental Synthesis Center, Annapolis, MD 21401 USA

## Abstract

Organic carbon accumulation in the sediments of inland aquatic and coastal ecosystems is an important process in the global carbon budget that is subject to intense human modification. To date, research has focused on quantifying accumulation rates in individual or groups of aquatic ecosystems to quantify the aquatic carbon sinks. However, there hasn’t been a synthesis of rates across aquatic ecosystem to address the variability in rates within and among ecosystems types. Doing so would identify gaps in our understanding of accumulation rates and potentially reveal carbon sinks vulnerable to change. We synthesized accumulation rates from the literature, compiling 464 rate measurements from 103 studies of carbon accumulated in the modern period (ca. 200 years). Accumulation rates from the literature spanned four orders of magnitude varying substantially within and among ecosystem categories, with mean estimates for ecosystem categories ranging from 15.6 to 73.2 g C m^−2^ y^−1^ within ecosystem categories. With the exception of lakes, mean accumulation rates were poorly constrained due to high variability and paucity of data. Despite the high uncertainty, the estimates of modern accumulation rate compiled here are an important step for constructing carbon budgets and predicting future change.

## Introduction

The stock and flux of organic carbon in the sediments of inland aquatic and coastal ecosystems comprises a substantial and active portion of the global carbon sink. For example, the organic carbon stock in coastal ecosystems is estimated to be more than half of ocean sediment carbon stock globally^1^ while annual lake and reservoir carbon accumulation is estimated to be similar to that of the global oceans^2,3^. The degree of human influence on the rate of organic carbon accumulation in inland aquatic and coastal ecosystems is also quite substantial. Anthropogenic drivers such as eutrophication, damming, sea level rise and climate change are actively modifying the rate of organic carbon accumulation in aquatic habitats^4–7^. While there is a desire to understand future changes to aquatic carbon accumulation in the context of anthropogenic effects, it is still unclear if our current estimates of global organic carbon accumulation in aquatic habitats are well constrained. Typically, investigations into organic carbon accumulation rates in aquatic ecosystems have been limited in scope to specific ecosystem categories or groupings (e.g. marsh, seagrass, lake; refs^8–10^). Mean estimates of carbon accumulation rates within an ecosystem category are then used to directly extrapolate mean continental or global scale organic carbon accumulation for that ecosystem^2,11–13^. In some instances, these globally scaled-up estimates have been used to establish carbon credit markets^14,15^ and suggest conservation and restoration as a marketable climate change mitigation strategy^16–18^. While these are promising avenues of research, we argue that there is still large uncertainty at the site level^19,20^, within ecosystem categories, and among aquatic ecosystems about aquatic organic carbon accumulation rates. To date, there has not been a systematic synthesis of organic carbon accumulation rates among all aquatic ecosystems. In addition to providing a better context for aquatic carbon accumulation rates and detection of broader patterns, a systematic synthesis of accumulation rates would also inform conservation and restoration initiatives and reveal research needs.

Comparing carbon accumulation estimates for aquatic ecosystems is hampered by a lack of standardized methods and definition of carbon accumulation. Varying interpretation as to what constitutes accumulation in aquatic ecosystems has resulted in a broad range of values reported as organic carbon accumulation in the literature. In particular, defining the time scale for carbon accumulation measurement is vital. For example, combining sedimentation rates at the scale of weeks and down-core accumulation rates measured at the scale of decades conflates two times scales for measuring organic carbon accumulation and can inflate mean estimates. Similarly, including organic carbon recently deposited at the sediment surface that has not undergone degradation with deeper, more permanently buried carbon biases long term estimates of accumulation rates^4^. A comparison of carbon accumulation rates among studies and ecosystem categories must account for the variety of methods used to estimate aquatic carbon accumulation to avoid drawing spurious conclusions about the magnitude of rates and sinks.

We performed a literature synthesis of modern era (ca 200 years or less) organic carbon accumulation rates among coastal and inland aquatic ecosystems. The goal of the synthesis was to 1) characterize the current knowledge regarding rates of aquatic organic carbon accumulation; 2) identify ecosystems where rates are poorly documented or have poor global representation; 3) determine if there are substantial differences in organic carbon accumulation among aquatic ecosystems, and; 4) examine broad-scale patterns in accumulation rates. We specifically focused on coastal and inland aquatic habitats and excluded the open ocean as depth, chemistry, and circulation make comparisons to the open ocean difficult^21^. The data included in the synthesis were restricted to published studies of modern era organic carbon accumulation rates (ca. 200 years or less) with direct measurements of accumulation in the sediment (see “Methods” for further description). We used a hierarchical Bayesian model to incorporate the reported mean and uncertainty in organic carbon accumulation rate from each reviewed study in order to estimate the overall carbon accumulation rate for various ecosystem categories (lake, reservoir, inland wetland, coastal wetland, lagoon, mangrove, continental shelf) and ecosystem characteristics (salinity and inundation). The resulting posterior distributions provide a valuable synthesis of existing data on aquatic carbon accumulation.

## Accumulation Rate by Ecosystem Category

**Figure 1:**
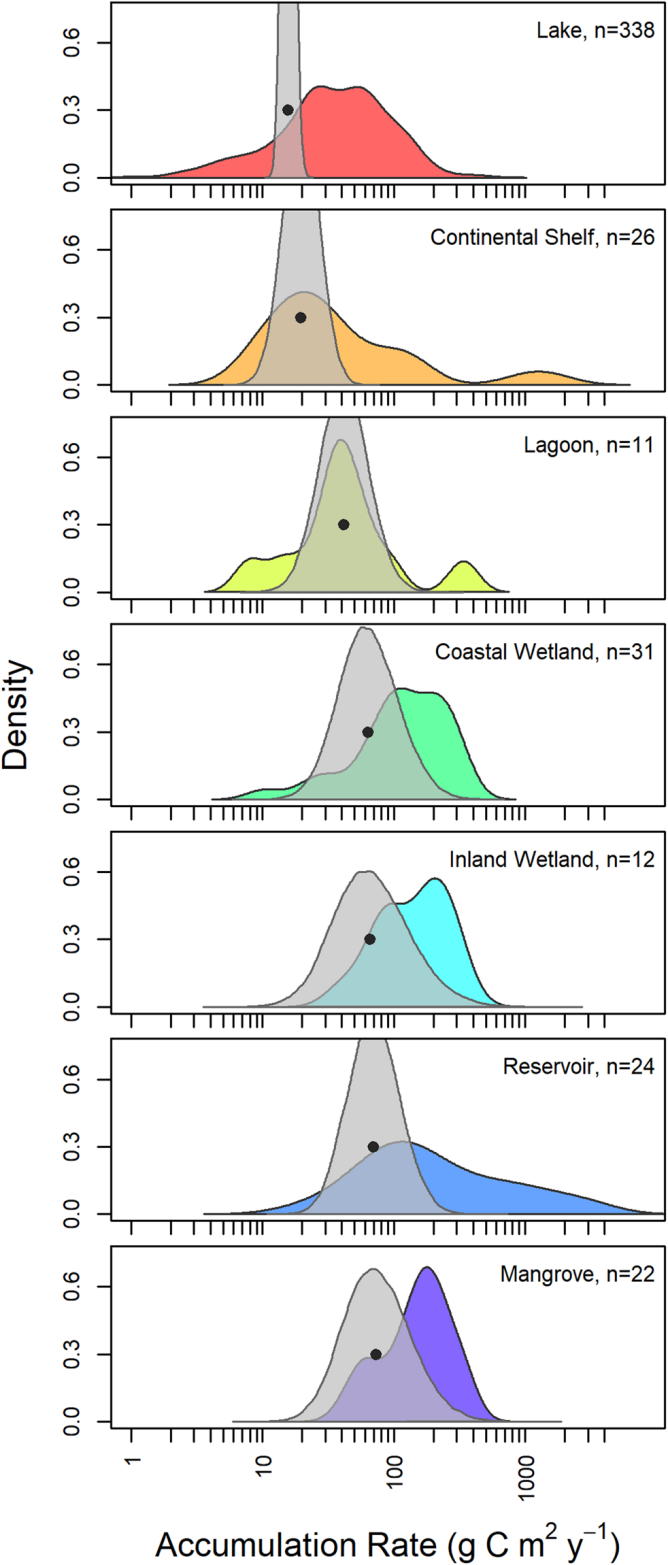
Estimate of the mean organic carbon accumulation rate in seven ecosystem categories. The colored distributions are the measurements of carbon accumulation from the literature. The gray distributions are the Bayesian posterior estimate of the mean accumulation rate. The median value of the posterior estimate for each ecosystem accumulation rate is denoted with a black circle.

There was a large amount of variability in reported organic carbon accumulation rates within the seven aquatic ecosystem categories but not among categories (Fig. 1; additional details of ecosystem categories can be found in Table S1). Within an ecosystem category, measurements of organic carbon accumulation spanned one to three orders of magnitude (Table 1) with a high degree of overlap among the distributions. Comparing the ranges among all pairings of ecosystem categories in Table 1, the average overlap in the range of accumulation rate distributions was 86.4% (Fig. 1). The data ranges for the continental shelf and reservoir ecosystems categories had the lowest degree of overlap with other ecosystem categories due to measurements of accumulation that exceeded 1000 g C m^−2^ y^−1^ in both categories. Despite these high rates, no single data distribution by ecosystem category was distinguished as having substantially higher or lower carbon accumulation rate measurements.

**Table 1:** The range of organic carbon accumulation rates by ecosystem type and the median and 95% credible intervals summary of the Bayesian posterior distributions of the mean organic carbon accumulation rate.

Category	Reported accumulation rate		Posterior distribution of mean accumulation rate		
	Minimum	Maximum	2.5%	*50%*	97.5%
Lake	1.1	479	13.1	*15*.*6*	18.9
Continental Shelf	7.54	1600	10.6	***19***.***6***	35.6
Lagoon	7.9	340	18.1	***41***.***4***	96.9
Coastal Wetland	10.2	335.8	24.1	***62***.***9***	186.8
Inland Wetland	42.3	306.3	20.0	***66***.***2***	281.1
Reservoir	20.3	2862	29.8	***69***.***9***	173.1
Mangrove	43.5	350	25.5	***73***.***2***	248.0
Permanent Inundation	1.1	2862	14.6	***17***.***3***	20.8
Seasonal Inundation	42.3	243	13.6	***57***.***7***	399.5
Tidal Inundation	7.5	350	26.3	***68***.***2***	165.2
Fresh water	1.1	2862	14.6	***17***.***6***	21.4
Brackish water	7.5	350	16.2	***25***.***8***	53.4
Saline water	6.0	337	15.3	***23***.***4***	43.4

All accumulation rates are in units of g C m^−2^ y^−1^.

The Bayesian hierarchical model provided posterior probability distributions of the mean organic carbon accumulation rate for each ecosystem category. The uncertainty in the modeled mean accumulation rates by category overlapped substantially providing little support for large differences in mean carbon accumulation rates among ecosystem types (Fig. 1). However, there were differences among ecosystem categories in the medians of the posterior distributions of estimated mean accumulation. For example, the mean organic carbon accumulation rate in mangrove forests was estimated to be four-fold higher than lakes (Table 1). All of the posterior distributions were broad except for the lake category, which had a much narrower posterior distribution. The broadness of the posterior distributions reveals how poorly constrained the estimates are in some ecosystem categories due to limited data and high variability. Despite the broad distributions, the incorporation of error into the model and resultant mean organic carbon accumulation rates provide a significant step forward in assessing ecosystem specific organic carbon accumulation rate and the uncertainty.

Compared to previous estimates in the literature of mean carbon accumulation by ecosystem category, our estimates are generally lower (Table S2). For example, the median mangrove and coastal wetland accumulation rate estimates are less than half that of prior estimates^11–13^. Similarly, the median lake estimate is less than half of estimates based on modeling carbon accumulation in United States lakes and reservoirs^22^, but similar to other studies encompassing North American lakes^4^ with direct measurements of carbon accumulation. Our estimates of lagoon organic carbon accumulation rate are only half of some earlier estimates which included sedimentation rates as measures of accumulation^11,12^, but similar to more recent values reported in the literature^16^. Mean estimates of reservoir organic carbon accumulation were 1–2 orders of magnitude greater^2,23,24^ than the mean estimate presented here. The mean accumulation rates estimated for continental shelf ecosystems is also similar to some^5^ but not all^10^ recent estimates. The discrepancies for this ecosystem type seem to largely be due to study methods.

Our estimates of carbon accumulation are likely lower for two reasons: 1) the restricted subset of studies included in our estimate due to the stringent inclusion criteria, and 2) explicit consideration of uncertainty in the estimate of mean accumulation rates. By only comparing studies that met strict criteria, defined *a priori*, we summarized data on longer-term carbon accumulation and did not include recent sedimentation fluxes which bias estimates upward. It is also possible that the search criteria excluded studies included in previous reviews; however, we reviewed a broad set of studies (see Supplementary Information). Additionally, the higher measurements of accumulation rate in the data set also had a higher degree of uncertainty associated with them. In the Bayesian model, both rare and uncertain measurements are less influential in estimating the mean carbon accumulation rate (a benefit of the Bayesian analysis). In order to test the robustness of this assumption, we also calculated the mean excluding uncertainty. Excluding information on the uncertainty of carbon accumulation within ecosystem categories resulted in slightly higher mean carbon accumulation estimates (Figs S2 and S3).

**Figure 2:**
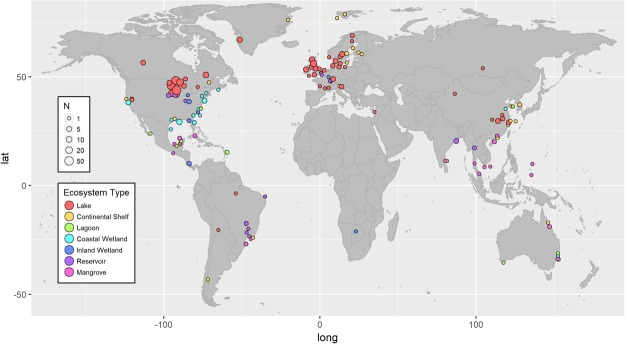
The geographic distribution of organic carbon accumulation measurements gathered from the literature. The color of points corresponds to ecosystem type and the size of points corresponds to the number of individual ecosystems measured in that location. Individual ecosystem locations were aggregated to a grid and points offset in order to show ecosystem type and global distribution; see supplemental table for precise locations.

It is important to note that the estimates of mean carbon accumulation rate presented here are the means for ecosystems that have been measured and not the actual global mean accumulation rates. For example, lakes had the greatest number of reported carbon accumulation measurements (Fig. 1) using our strict study inclusion criteria (detailed in Fig. S3). However, the geographic distribution was mainly restricted to temperate lakes in Europe and the Midwestern United States (Fig. 2). There were fewer measurements of carbon accumulation in boreal and arctic lakes despite the high abundance of lakes occurring in these regions^25^ as well as few measurements in tropical lakes. Similarly, despite the near-global distribution of salt marsh^26^, measurements of carbon accumulation in coastal wetlands were largely made in the eastern United States. Based on the differences in the geographical distribution of all aquatic ecosystems and the measured population of that ecosystem type, it is unlikely that the mean carbon accumulation rates presented here are equivalent to the true global mean carbon accumulation rates. However, the values from this analysis are currently the best estimate for comparing carbon accumulation rates in aquatic ecosystems.

## Patterns in Accumulation Rates

Organic carbon accumulation in coastal and inland aquatic ecosystems is subject to direct and indirect modification by human activities. Despite the high uncertainty in accumulation estimates in most of the ecosystem categories we can still use the literature synthesis and model to generate broad hypotheses concerning the consequences of anthropogenic influence on accumulation rates. The loss of mangroves^27^ and coastal wetlands^28^ due to sea-level rise or human development will potentially shift these locations high in organic carbon accumulation to lower rate sites similar to continental shelf habitats. Based on this synthesis, this could result in an approximately threefold decline in the organic carbon accumulation rate (Table 1). Conversely, the number of impoundments is increasing across the globe^29,30^. These ecosystems have higher organic carbon accumulation rates compared to other inland aquatic habitats (Table 1). While the data presented here indicate that increasing reservoir prevalence is likely to result in increased organic carbon accumulation, the analysis also reveals a lack of data from lower latitudes where reservoirs surface area is greatly increasing.

**Figure 3:**
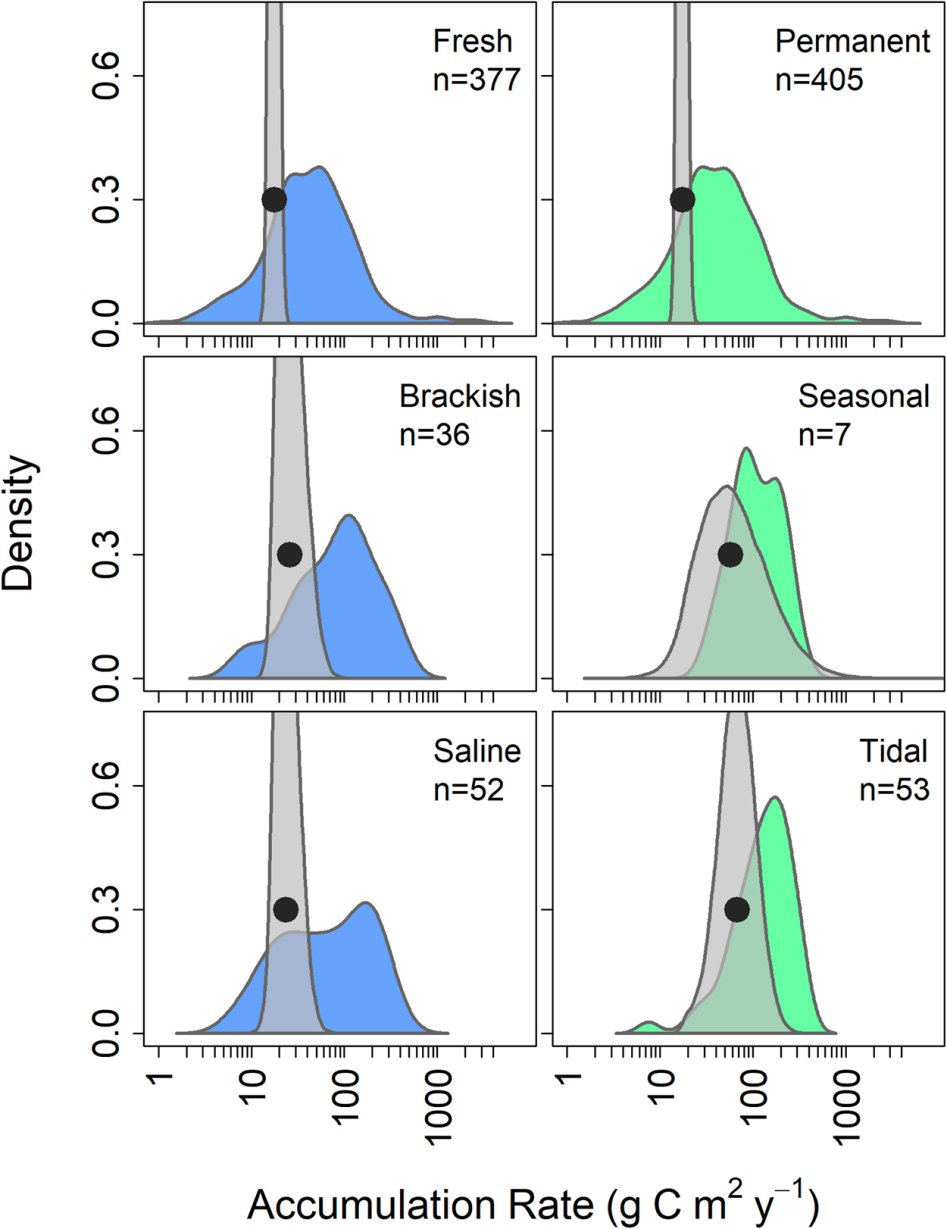
Estimate of the mean organic carbon accumulation rates categorized by inundation frequency and salinity. The colored distributions are the carbon accumulation measurements from the literature with the number of sites noted above each distribution. The gray distributions are the Bayesian posterior estimate of the mean carbon accumulation rate for the category. The median value of the posterior estimate is denoted with a black circle. The left column contains the distributions for the salinity categories and the column to the right contains the distributions for the inundation categories.

In addition to evaluating patterns in accumulation rate among ecosystem categories, we also evaluated two generic aquatic ecosystem characteristics to examine broader patterns in rates. Sediment redox conditions are a driver of carbon accumulation rates^5,31,32^ and vary between fresh and saline waters. Compared to inland waters, saline coastal sediments are generally high in sulfate which is a more favorable electron acceptor compared to those often available in freshwaters^33^. As few studies reported sediment redox conditions explicitly, we used three salinity categories as a proxy for broad differences in sulfate availability (Fig. 3). Despite a slightly higher mean accumulation rate in saline and brackish ecosystems compared to freshwater, there was little difference in the mean estimated accumulation rate for each salinity category (Table 1). While this result suggests that assumed differences in sulfate availability are not a main driver of carbon accumulation rates at a broad scale, sediment redox conditions, particularly oxygen concentration^31,34^, are likely still an important driver of carbon accumulation at a local scale (e.g.^35,36^).

The frequency of inundation is another generic aquatic ecosystem characteristic that could influence carbon accumulation. Inundation frequency influences the physical delivery of carbon to the sediments, including exogenous organic carbon. Tidal ecosystems had a mean carbon accumulation rate 3.9x higher than ecosystems that are permanently inundated (Fig. 3). The higher accumulation rate in coastal tidal ecosystems can partially be attributed to the continual import of carbon from adjacent ecosystems such as rivers^37^ and offshore sediments. Additionally, the estimate of carbon accumulation in tidal ecosystems is mainly based on vegetated “blue carbon” ecosystems (i.e. coastal wetlands and mangroves). There were few measurements of accumulation rates in unvegetated tidal ecosystems (Table S1). Resuspension and deposition in unvegetated tidal areas could potentially reduce overall carbon accumulation^38–40^, but there are currently not comparable data to evaluate this hypothesis.

Other drivers of organic carbon accumulation also likely act at a broader scale than individual ecosystems. Sediment conditions such as temperature^41^, the redox environment^31,42^, organic matter lability^43,44^, bed morphometry^45–47^ and sediment focusing^48^ alter the rate of carbon accumulation in aquatic ecosystems. These drivers could not be evaluated among ecosystem types in this analysis because most studies did not report the relevant information. For example, while numerous studies of lake organic carbon accumulation provide some metric of human impact on lake ecosystem function such as eutrophication^4,8^, the majority of other studies did not report this information. As such, given the dominance of lake accumulation rates in our data set, any comparison of inter-ecosystem drivers would have been informed largely by the pattern in lakes.

## Recommendations for Future Research

Overall, 80% of the values we summarized were from inland water habitats, dominated by lakes, while only 20% were from coastal marine ecosystems. Although some aquatic ecosystem types had well constrained estimates of mean organic carbon accumulation, many such as inland wetlands and mangroves were poorly constrained due to limited data and high variability (Fig. 1; Table 1). Even the ecosystem categories such as lakes that did have a well constrained estimate of mean carbon accumulation lacked globally-representative measurements (e.g., boreal and tropical lake data) to inform the estimate. Measurements of accumulation using methods not included in this study due the criteria for inclusion (Fig. S3) may exist for these regions and would be informative. However, additional and geographically diverse measurement efforts are still needed for all aquatic ecosystems in order to draw robust conclusions about global organic carbon accumulation rates.

Future studies and syntheses of carbon accumulation rates, particularly those including multiple aquatic ecosystems, should focus on the mechanisms explaining variation in carbon accumulation rates. A deeper understanding of the mechanisms that control aquatic carbon accumulation rates regardless of ecosystem type will allow for more robust global predictions of current carbon accumulation rates and how those rates are likely to be modified in the future. Additionally, investigators need to begin reporting additional information such as water depth, the redox environment, nutrient concentrations, and watershed land use information to provide context for carbon accumulation measurements.

Based on this systematic literature review, it is clear that more data are needed to constrain carbon accumulation rates in aquatic ecosystems. Clearly, there is substantial variability in carbon accumulation rates within ecosystem categories with a high degree of overlap among systems. The high degree of variability in carbon accumulation measurements within ecosystem categories indicates that estimates of global annual carbon accumulation are highly uncertain for most ecosystems categories. For example, the global mean annual carbon accumulation rate can be calculated using the results in Table 1 and the global coverage of ecosystem categories. For mangroves, the global mean annual carbon accumulation is estimated as 11.7 Tg C y^−1^ based on coverage from ref.^49^. However, the high uncertainty in the mean mangrove accumulation rate yields estimates ranging from 4.1 to 39.7 Tg C y^−1^ which is 35 to 339% of the mean. Conversely, the global mean annual carbon accumulation rate for lakes is 78 Tg y^−1^ based on global area^25^. The range for this well constrained category is 65.5 to 94.5 Tg y^−1^, or only 84 to 121% of the mean estimate.

Until additional data representative of the variability in conditions and geographic location are available, upscaling organic carbon accumulation rates for most ecosystem categories should be done with caution. Additionally, given the larger degree of uncertainty in some ecosystem categories, we do not recommend the use of up-scaled estimates of carbon accumulation from the limited data available for broad scale carbon sink management. If up-scaled carbon accumulation estimates are used as a basis for carbon management programs, we recommend careful consideration of the methods used to define and measure what constitutes “accumulation” to ensure compatibility with carbon sink management goals.

## Methods

A systematic literature review was performed using the ISI Web of Knowledge database and completed on 15 Sept 2016. In order to include relevant studies from a diversity of disciplines, we used six truncated phrases to search for studies on carbon accumulation (“c burial” OR “carbon burial” OR “c accumulat*“ OR “carbon accumulat*“ OR “organic matter accumulation” OR “organic matter accumulat*“). These six phrases were used in combination with the truncated ecosystem names presented in Table S1. The search was refined to peer-reviewed articles in English language journals. In total, 3,853 papers were returned and evaluated for inclusion based on review of the title and abstract, and methods.

Stringent guidelines for inclusion in the synthesis were developed to ensure estimates of carbon accumulation rates were comparable across ecosystem types. In order to be included in the analysis, a carbon accumulation rate measurement had to be derived from direct measurements of the organic carbon in the sediment (e.g. sediment trap measurements were excluded) and not a modeled value from a carbon mass balance. The method for dating the sediment profile also had to be derived from direct measurements including radionuclides, varve counting, or known event horizons. Additionally, the carbon accumulation rate measurements had to be modern, defined in our analysis as spanning the most recent ca. 200 years. As the focus on the literature synthesis was on recent accumulation rates, sediments dated with radiocarbon methods were not included because they are not comparable with other radionuclide measurements with a shorter half-life. Studies that measured carbon accumulation rates at time scales less than 10 years were excluded to prevent inflated rate estimates based on recently deposited organic carbon that may not have undergone significant diagenesis. Carbon accumulation measurements from experimental treatments were excluded; however, rates from restored ecosystems (*n* = 9) were included if the restoration occurred more than 10 years prior.

As this was a literature synthesis and not original measurements, we accepted the original, peer-reviewed data analysis methods of the investigators. This includes lack of correction of sediment focusing^48^, the radioisotope dating model that was used, and the method of statistically summarizing the accumulation rate when multiple cores were taken. The variability in methods and data analysis introduces some amount of uncertainty in our analysis. Despite this, we deferred to the original investigators decisions whenever possible as experts in their study locations and methods. We also included any reported uncertainty at the study level in our analysis. The standard deviation of each system’s carbon accumulation rate was recorded when available (194 of the 464 rate measurements). When not provided, we estimated standard deviation by multiplying the mean ecosystem-specific coefficient of variation by the reported accumulation rate measurement (see Supplementary Information for more details).

We were most interested in comparing the carbon accumulation rates in aquatic ecosystem types with the greatest human impact. As such, we limited the literature synthesis to inland and coastal aquatic systems with frequent (daily to seasonal) or permanent surface inundation thereby excluding river flood plains, fens, and bogs. While we included “seas” as an ecosystem type in our search terms in order to identify potentially relevant studies in inland and coastal seas, we discarded any marine carbon accumulation measurements from depths greater than 200 m to generally make the analysis based on areas above the pycnocline of the open ocean. For more information on the literature synthesis study inclusion criteria, see Figure S3. Additionally, some ecosystem categories had few measurements based on our stringent inclusion guidelines (e.g. fjords) and were therefore grouped into a larger, hydrologically relevant category for the Bayesian analysis. Information on grouped categories is provided in Table S1.

We used a hierarchical Bayesian model to estimate the mean carbon accumulation rate for each ecosystem type and ecosystem characteristic among types (e.g. salinity). We selected a hierarchical Bayesian approach because it allows us to simultaneously incorporate each reviewed study’s mean and associated uncertainty in the estimate of the system group’s carbon accumulation rate. In doing so, high estimates that are highly uncertain are less influential on the estimated mean. Further, the resulting posterior estimates for the means can be updated with information from future carbon accumulation studies. Weakly informative priors were used for the mean (uniform distribution from 0.1 to 10000) and standard deviation (gamma distribution with shape parameters of 0.001). The minimum in the prior for the mean was selected based on the theoretical detection limit of the carbon accumulation estimation methods and the maximum was selected based as an order of magnitude higher than rates reported in the literature. We selected weakly informative priors because there is no suitable existing information on the system group means beyond the data compiled for this study. When the data for a system group do not overcome a weakly informative prior, it indicates that the existing data does not contain sufficient information to overwhelm even weak prior information. Importantly, this result supports the need for additional information on carbon accumulation in these systems. Each Bayesian model was implemented in JAGS using the R package “rjags” (Plummer 2013). For each model, we ran three chains for 300,000 iterations, with a burn in of 100,000 iterations. Convergence was checked with trace plots and the Gelman-Rubin convergence statistic^50^.

## Electronic supplementary material

Supplementary Information

Dataset 1
