# Supplementary material for: A synthesis of modern organic carbon accumulation rates in coastal and aquatic inland ecosystems
Source: Sci Rep. 2018 Oct 24;8:15736. doi: 10.1038/s41598-018-34126-y (PMC6200792; doi:10.1038/s41598-018-34126-y)
Supplement: Supplementary file 1 — Supplementary Information [file 41598_2018_34126_MOESM1_ESM.docx]

**SUPPLEMENTARY INFORMATION**:
***A synthesis of modern organic carbon accumulation rates in
coastal and aquatic inland ecosystems***

Grace M. Wilkinson, Alice Besterman, Cal Buelo, Jessica Gephart, Michael L. Pace

***Details of study selection and inclusion***

In order to make the most robust and direct comparison of organic carbon accumulation rates among ecosystem categories, stringent guidelines for study inclusion were developed for this comparative analysis. Using the ecosystem category search terms in Table S1 combined with the organic carbon search terms ("c burial" OR "carbon burial" OR "c accumulat*" OR "carbon accumulat*" OR "organic matter burial" OR "organic matter accumulat*") returned 3,853 studies. Each study was evaluated using the decision tree criteria in Figure S3. Studies that met the inclusion criteria (see Supplementary data file, metadata in Table S3) were assigned to an ecosystem, salinity, and inundation frequency category based on information reported in each study.
 In some instances, organic carbon burial rates needed to be calculated from the measurements reported in the study. For example, some studies reported sediment accumulation rates and the organic carbon content of the sediment as a percentage of the total sediment. These two values were then multiplied together to calculate organic carbon accumulation rate. Some studies only included organic carbon accumulation data in figures. For these studies we used the application WebPlotDigitizer (http://arohatgi.info/WebPlotDigitizer/app/) to extract carbon accumulation rates. In order to test if the data extraction tool was accurate, we compared our extracted estimate for studies that used both reported the carbon accumulation rate and presented in the information in a figure. The units for all rates and errors were converted to the same unit using the equations in Table S4.

***Bayesian model error estimation*** Our estimates of carbon accumulation include data on each system’s mean and standard deviation. When information on the standard deviation is not included in the model, the estimated mean carbon accumulation for each category (i.e. ecosystem, salinity, or inundation group) matches the mean of the distribution of means. These values are slightly higher than the carbon accumulation estimates reported in this study. This can be explained by systems with higher means generally having higher within system variability in carbon accumulation. However, including data on each system’s standard deviation allows us to incorporate this variability in carbon accumulation in a single system into each group’s overall carbon accumulation estimate.
 Of the 464 observations, 194 reported a standard deviation. As a result, we interpolated the missing standard deviations by multiplying the mean ecosystem-specific coefficient of variation by the reported accumulation rate measurement. An alternative approach is to model the missing data within a Bayesian model. We compared the results of the two modeling approaches: 1) modeling the missing standard deviations as a linear function of the means (SI Fig 1 & 2) and 2) interpolating based on the mean ecosystem-specific coefficient of variation (presented in the main text). We found that although the carbon accumulation estimates were generally slightly lower by modeling the standard deviations, the overall pattern and conclusion about the high uncertainty of accumulation estimates held.

***Figure S1.*** Posterior distribution of the mean organic carbon accumulation rate by ecosystem category without variance assigned to studies that did not report an error estimate. The colored distributions are the measurements of carbon accumulation from the literature. The gray distributions are the Bayesian posterior estimate of the mean accumulation rate. The median value of the posterior estimate for each ecosystem accumulation rate is denoted with a black circle.

***
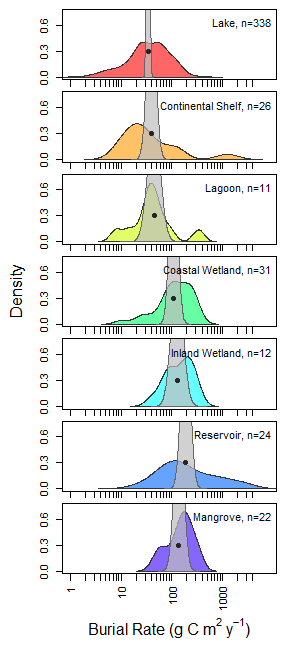
***

***Figure S2.*** Posterior distribution of the mean organic carbon accumulation rate by salinity and inundation frequency category without variance assigned to studies that did not report an error estimate. The colored distributions are the measurements of carbon accumulation from the literature. The gray distributions are the Bayesian posterior estimate of the mean accumulation rate. The median value of the posterior estimate for each ecosystem accumulation rate is denoted with a black circle.

***
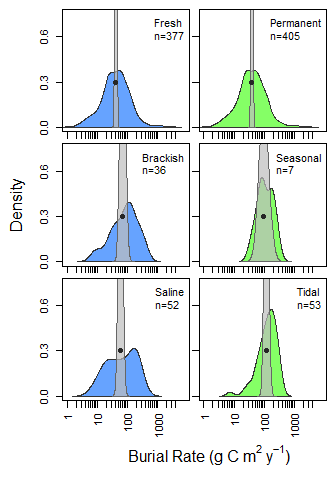
***

***Figure S3.*** Flowchart of decision process for study inclusion in this analysis using the search terms in Table S1.

***
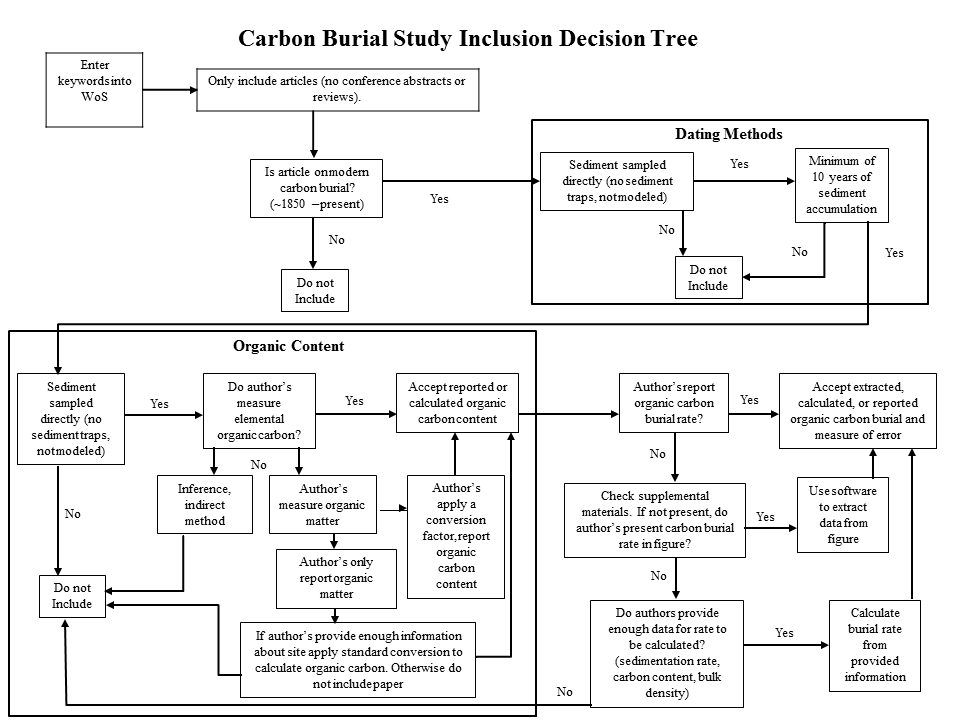
***

|  | **Classification** | **Ecosystem** | **Keyword Search Term** | **Studies Returned** | **Studies Included** | **# of Sites** |
| --- | --- | --- | --- | --- | --- | --- |
| **Inland Waters** | Lake | Lake | Lake* | 338 | 26 | 338 |
|  | Reservoir | Reservoir, Pond | Reservoir*, Pond* | 177 | 10 | 24 |
|  | Inland Wetland | Inland Wetland | Wetland* OR Marsh* OR Swamp* | 478 | 9 | 12 |
|  | -- | River | River* | 327 | 0 | -- |
|  | -- | Stream | Stream* | 89 | 0 | -- |
|  | -- | Spring | Spring* | 77 | 0 | -- |
| **Coastal Waters** | Lagoon | Lagoon, Seagrass | Seagrass*, Lagoon* | 85 | 7 | 11 |
|  | Mangrove | Mangrove | Mangrove* | 72 | 14 | 22 |
|  | Coastal Wetland | Coastal Wetland | Wetland* OR Marsh* OR Swamp* | 478 | 16 | 31 |
|  | Continental Shelf | Delta, Fjord, Estuary, Continental Shelf | Delta*, Fjord*, Estuar*, Continental Shel*, Bay*, Gulf*, Sea*, Strait*, Bight*, Sound*, Cove*, Cape* | 2003 | 21 | 26 |
|  | -- | Intertidal | Intertidal* | 31 | 0 | -- |
|  | -- | Mudflat | Mudflat* | 13 | 0 | -- |
|  | -- | Beach | Beach* | 7 | 0 | -- |
|  | -- | Coral Reef | Coral Reef* | 15 | 0 | -- |

***Table S1.*** Summary of the literature review and ecosystem type classification. Note while many studies were returned by the search terms used relatively few had direct measures of carbon accumulation rates that met the criteria of this study.

***Table S2.*** Examples of other synthesis or modeling studies of carbon accumulation in various aquatic ecosystems. The majority of these studies were not included in the analysis presented in this paper as they did not meet the criteria in Figure S3, mainly that the rates were either not measured directly or were not modern (ca. 200 years old). Organic carbon accumulation rates are in g C m^-2^ y^-1^.

| ***Ecosystem Category*** | ***First Author*** | ***DOI*** | ***Carbon Accumulation Rate*** | ***Summary of method*** |
| --- | --- | --- | --- | --- |
| Continental Shelf | Bauer et al. (2013) | 10.1038/nature12857 | 13 | literature values of the global accumulation rate |
| Continental Shelf | Duarte et al. (2005) | 10.5194/bg-2-1-2005 | 17 | Geometric mean of literature review values |
| Estuary (Continental Shelf) | Duarte et al. (2005) | 10.5194/bg-2-1-2005 | 45 | Geometric mean of literature review values |
| Fjords (Continental Shelf) | Smith et al. (2015) | 10.1038/ngeo2421 | 54 | Data assembled from literature and other sources, where all information was not available author's calculated rates by using approximate values |
| Lakes | Alin et al. (2007) | 10.1029/2006GB002881 | 1-20 | Synthesis of literature values for global large lakes |
| Lakes | Einsele et al. (2001) | 10.1016/S0921-8181(01)00105-9 | 2-40 | Literature review and modeling, values from Table 5 therein |
| Lakes | Dean and Gorham (1998) | http://digitalcommons.unl.edu/usgsstaffpub/299 | 5-72 | literature review + original measurements |
| Lakes | Kortelainen e tal. (2004) | 10.1111/j.1365-2486.2004.00848.x | 0.2-8.5 | Finnish lake Holocene organic carbon accumulation rates |
| Lakes | Katowski et al. (2011) | 10.1029/2010GB003874 | 5.6 | Modeled carbon accumulation rates in Europe |
| Mangrove | Chmura et al. (2003) | 10.1029/2002GB001917 | 210 | Average of 26 studies with 122 sites for tidal saline wetlands. No differences between mangroves and salt marshes, so one mean value for the two systems produced |
| Mangrove | Lovelock et al. (2010) | 10.1007/s10021-010-9329-2 | 367 | Direct measurements collected from Firth of Thames in New Zealand |
| Mangrove | Duart et al. (2005) | 10.5194/bg-2-1-2005 | 139 | Geometric mean of literature review values |
| Mangrove | McLeod et al. (2011) | 10.1890/110004 | 226 | Average of data from other reviews + original measurements (including other reviews listed here) |
| Mangrove | Breithaupt et al. (2012) | 10.1029/2012GB004375 | 163 | Geometric mean of literature review values for centennial-scale accumulation, excluded short tern studies (1-2 years), also excluded mudflats |
| Reservoirs | Mulholland and Elwood (1982) | Tellus 34: 490-499 | 500 | Literature review and modeled value |
| Reservoirs | Dean and Gorham (1998) | http://digitalcommons.unl.edu/usgsstaffpub/299 | 400 | literature review + modeling |
| Reservoirs | Downing et al. (2008) | doi:10.1029/2006GB002854 | 2000 | Extrapolation from measurements in numerous eutrophic impoundments |
| Salt Marsh (Coastal Wetland) | Chmura et al. (2003) | 10.1029/2002GB001917 | 210 | Average of 26 studies with 122 sites for tidal saline wetlands. Authors found no differences between mangroves and salt marshes so produced one mean value for the two systems |
| Salt Marsh (Coastal Wetland) | Duarte et al. (2005) | 10.5194/bg-2-1-2005 | 151 | Geometric mean of literature review values |
| Salt Marsh (Coastal Wetland) | McLeod et al. (2011) | 10.1890/110004 | 218 | Average of data from other reviews + some original measurements (including other reviews listed here) |
| Seagrass (Lagoon) | Duarte et al. (2005) | 10.5194/bg-2-1-2005 | 83 | Geometric mean of literature review values |
| Seagrass (Lagoon) | Kennedy et al. (2010) | 10.1029/2010GB003848 | 160-186 | net community production + allochthonous carbon in sediments. Determined allochthonous portion by applying stable isotopes to literature review values, net community production from Duarte 2010 |
| Seagrass (Lagoon) | McLeod et al. (2011) | 10.1890/110004 | 138 | Average of data from other reviews + some original measurements (including reviews listed here) |
| Seagrass (Lagoon) | Duarte et al. (2013) | 10.1016/j.ocecoaman.2011.09.001 | 53 | literature values of short-term accumulation (not radiocarbon; Pb-210 and other methods) |
| Seagrass (Lagoon) | Duarte et al. (2013) | 10.1016/j.ocecoaman.2011.09.001 | 58 | literature values of "long-term" rates (using radiocarbon) |

***Table S3.*** Description of data table column headers

| **Column Title** | **Variable** | **Type** | **Values** |
| --- | --- | --- | --- |
| **Ecosystem_Type** | ecosystem classification | character | e.g. “Lake” or “Lagoon” |
| **Site** | site of coring | character | e.g. “Station A” or “Station B” |
| **Location** | location of study | character | e.g. “Bay A” or “Weddell Sea” |
| **Country_Sea** | country or sea of study | character | the country or oceanic body of water where the study was performed |
| **Sample_Site_Description** | coring site description | character | description of core location such as “deepest point” or “fringe” |
| **First_Author** | last name | character | last name of the first author of the study |
| **Year** | publication year | numeric | year that the study was published |
| **DOI_Title** | DOI or Article Title | character | Digital Object Identifier for study or full title of the study for studies without a DOI |
| **Burial Rate** | carbon accumulation rate | numeric | reported or calculated organic carbon accumulation rate |
| **BR_Units** | aerial rate units | character | aerial units of the organic carbon accumulation rate, all converted to g C m^-2^ yr^-1^ |
| **Error_Estimate** | error of C accumultion estimates | numeric | reported estimated error on the carbon accumulation rate |
| **Error_Type** | type of calculated error | character | SD= standard deviation, SE= standard error, NR= not reported |
| **Burial_YearRange** | number of years encompassed in rate | numeric | either based on reported measurement interval or estimated based on radionucleotide method |
| **OC_Method** | method for measuring sediment organic carbon | character | the method or instrumentation used to measure organic carbon. LOI= loss on ignition, LOI to C = loss on ignition converted to organic C by authors in study or by the authors of this project. See notes for further information. TOC Analyzer = Total Organic Carbon Analyzer. Other methods are spelled out. |
| **IC_Correction** | if an inorganic carbon correction was applied | character | Yes= inorganic carbon corrected for or inorganic carbon was negligible, No= to correction was specifically not performed |
| **Sampling _Method** | method of sediment sampling | character | Core= a core of non-surficial depth was taken to measure organic carbon. Non-coring methods were excluded from these analyses. |
| **Dating_Method** | method of sediment dating | character | “Radionuclide”; “Stratigraphic marker”; “Known age”; “Radionuclide, varve” |
| **Radionuclide** | radioisotope used to date | character | radioisotope method used to date the sediment core; e.g. “Pb210” or “Cs137” |
| **Isotope_Model** | radioisotope model for sediment accumulation rate | character | method used to model sediment accumulation rate from radioisotope profiles; if left blank then either authors did not report OR authors used an unnamed method/procedure. See notes for further information. |
| **Sample_Depth** | water depth | numeric | water depth at the sediment sampling site in meters |
| **Latitude** | study site latitude | numeric | latitude, in decimal degrees, of the study site or location |
| **Longitude** | study site longitude | numeric | longitude, in decimal degrees, of the study site or location |
| **System_Size** | ecosystem size | numeric | reported system size in square kilometers |
| **Salinity_Category** | water salinity | character | “Saline”; “Brackish”; “Fresh” |
| **Inundation_Frequency** | frequency of water inundation | character | “Tidal”; “Permanent”; “Seasonal” = inundated annually for prolonged period; e.g. vernal pool |
| **Notes** | notes on study use | character | notes on the use of the study, assumptions, data manipulations, and other references with supplementary information on the ecosystem |

***Table S4.*** Equations used to convert units of carbon accumulation from reported units in the literature to g C m^-2^ y^-1^.

| **Original Units** | **Full Conversion** | **Simplified Conversion** |
| --- | --- | --- |
| mg C cm^-2^ yr^-1^ | $\frac{\text{mg C}}{\text{cm}^{2}\text{ yr}}\times\frac{\text{1 g}}{\text{1,000 mg}}\times\frac{\text{10,000 cm}^{2}}{\text{1 m}^{2}}=\text{ g C m}^{-2}\text{ yr}^{-1}$ | $\text{mg C cm}^{-2}\text{ yr}^{-1}\times\text{ 10}$ |
| mg C m^-2^ d^-1^ | $\frac{\text{mg C}}{\text{m}^{2}\text{ d}}\times\frac{\text{365 d}}{\text{1 yr}}\times\frac{\text{1 g}}{\text{1,000 mg}}=\text{ g C m}^{-2}\text{ yr}^{-1}$ | $\text{mg C m}^{-2}\text{ d}^{-1}\times\text{ 0.365}$ |
| g C cm^-2^ yr^-1^ | $\frac{\text{g C}}{\text{cm}^{2}\text{yr}}\times\frac{\text{10,000 cm}^{2}}{\text{1 m}^{2}}=\text{ g C m}^{-2}\text{ yr}^{-1}$ | $\text{g C cm}^{-2}\text{ yr}^{-1}\times\text{ 10,000}$ |
| g C 100 cm^-2^ yr^-1^ | $\frac{\text{g C}}{\text{100 cm}^{2}\text{yr}}\times\frac{\text{10,000 cm}^{2}}{\text{1 m}^{2}}=\text{ g C m}^{-2}\text{ yr}^{-1}$ | $\text{g C 100 cm}^{-2}\text{ yr}^{-1}\times100$ |
| kg C m^-2^ yr^-1^ | $\frac{\text{kg C}}{\text{m}^{2}\text{yr}}\times\frac{\text{1,000 g}}{\text{1 kg}}=\text{ g C m}^{-2}\text{ yr}^{-1}$ | $\text{kg C m}^{\text{-2}}\text{ yr}^{\text{-1}}\text{×1,000}$ |
| kg C ha^-1^ yr^-1^ | $\frac{\text{kg C}}{\text{ha yr}}\times\frac{\text{1,000 g}}{\text{1 kg}}\times\frac{\text{1 ha}}{\text{10,000 m}^{2}}=\text{ g C m}^{-2}\text{ yr}^{-1}$ | $\text{kg C ha}^{-1}\text{ yr}^{-1}\times\text{ 0.1}$ |
| Mg C ha^-1^ yr^-1^ | $\frac{\text{Mg C}}{\text{ha yr}}\times\frac{\text{1 ha}}{\text{10,000 m}^{2}}\times\frac{\text{1,000,000 g}}{\text{1 Mg}}=\text{ g C m}^{-2}\text{ yr}^{-1}$ | $\text{Mg C ha}^{-1}\text{ yr}^{-1}\times\text{ }100$ |
| mmol C m^-2^ d^-1^ | $\frac{\text{mmol C}}{\text{m}^{2}\text{ d}}\times\frac{\text{365 d}}{\text{1 yr}}\times\frac{\text{1 mol}}{\text{1000 mmol}}\times\frac{\text{12.01 g}}{\text{1 mol}}=\text{ g C m}^{-2}\text{ yr}^{-1}$ | $\text{mmol C m}^{-2}\text{ d}^{-1}\times4.38365$ |
| mmol C m^-2^ yr^-1^ | $\frac{\text{mmol C}}{\text{m}^{2}\text{ yr}}\times\frac{\text{1 mol}}{\text{1000 mmol}}\times\frac{\text{12.01 g}}{\text{1 mol}}=\text{ g C m}^{-2}\text{ yr}^{-1}$ | $\text{mmol C m}^{-2}\text{ yr}^{-1}\times0.01201$ |
| mol C m^-2^ yr^-1^ | $\frac{\text{mol C}}{\text{m}^{2}\text{ yr}}\times\frac{\text{12.01 g}}{\text{1 mol}}=\text{ g C m}^{-2}\text{ yr}^{-1}$ | $\text{mol C m}^{-2}\text{ yr}^{-1}\times12.01$ |
